# Diverse rhizospheric *Bacillus* are required for protection against a leaf pathogen

**DOI:** 10.1093/ismejo/wraf134

**Published:** 2025-07-01

**Authors:** Amal Ghosheh, Moshe Alon, Shay Kenneth-Mordoch, Ziv Kleinman, Marnix H Medema, Omri M Finkel

**Affiliations:** Department of Plant & Environmental Sciences, The Alexander Silberman Institute of Life Sciences, The Hebrew University of Jerusalem, Edmond J. Safra Campus—Givat Ram, Jerusalem 9190401, Israel; Department of Plant & Environmental Sciences, The Alexander Silberman Institute of Life Sciences, The Hebrew University of Jerusalem, Edmond J. Safra Campus—Givat Ram, Jerusalem 9190401, Israel; Department of Plant & Environmental Sciences, The Alexander Silberman Institute of Life Sciences, The Hebrew University of Jerusalem, Edmond J. Safra Campus—Givat Ram, Jerusalem 9190401, Israel; Department of Plant & Environmental Sciences, The Alexander Silberman Institute of Life Sciences, The Hebrew University of Jerusalem, Edmond J. Safra Campus—Givat Ram, Jerusalem 9190401, Israel; Bioinformatics Group, Wageningen University, Droevendaalsesteeg 1, 6708 PB Wageningen, The Netherlands; Institute of Biology Leiden University, Sylviusweg 72, 2333 BE Leiden, The Netherlands; Department of Plant & Environmental Sciences, The Alexander Silberman Institute of Life Sciences, The Hebrew University of Jerusalem, Edmond J. Safra Campus—Givat Ram, Jerusalem 9190401, Israel

**Keywords:** plant microbiome, synthetic communities, biocontrol

## Abstract

The microbiota plays a crucial role in protecting plants from pests and pathogens, as experimental disruptions to the microbiota cause plants to succumb to otherwise asymptomatic infections. To understand how microbial plant defense is deployed, we applied a complex and tractable plant–soil–microbiome microcosm. This system, consisting of *Arabidopsis* plants and a 150-member bacterial synthetic community, provides a platform for the discovery of novel bacterial plant-beneficial traits, under a realistically complex microbial community context. To identify which components of the plant microbiota are critical for plant defense, we deconstructed this microcosm top-down, removing different microbial groups from the community to examine their protective effect on the plant when challenged with the leaf pathogen *Pseudomonas syringae* pv. tomato DC3000. This process of community deconstruction revealed a critical role for the genus *Bacillus* in protecting the plant from infection. Using plant RNA-seq and bacterial co-culturing experiments, we demonstrated that *Bacillus-*provided plant protection is independent of plant immune system activation. We also show that the level of plant protection is strongly dependent on the diversity of the protective inoculum. Applying inocula with high within-genus diversity offers a significant improvement to current biocontrol strategies.

## Introduction

In healthy plants, many potentially pathogenic microbes are unable to manifest disease [1, 2]. This is partly due to the plants’ sophisticated immune system but at least equally important is the critical defense provided by the plant microbiota, which play an essential role in maintaining plant health and resilience [3–7]. When plants are grown without their associated microbiome, they exhibit a complete mortality rate when faced with even non-aggressive pathogens [2]. The microbiome’s bacteria suppress plant disease by several means: directly antagonizing pathogens [8], niche competition [9], or interference with pathogen virulence [10]. The plant microbiome has also been shown to bolster the plant’s immune system, inducing a state of enhanced defensive readiness known as induced systemic resistance (ISR) [11, 12].

The microbiome, as an assembly of thousands of species, each with distinct plant and inter-microbial interactions, presents a challenge in identifying those interactions that are most protective. Infected plants were shown to recruit taxa such as *Streptomyces* in the defense against *Ralstonia solanacearum* in tomatoes [13], *Chitinophaga* and *Flavobacterium* against *Rhizoctonia solani* in sugarbeet [14], and *Stenotrophomonas, Xanthomonas*, and *Microbacterium* against *Hyaloperonospora arabidopsidis* in *Arabidopsis* [15, 16]. These examples commonly reveal a pathogen-specific enrichment of bacterial taxa, yet they may overlook the continuous baseline defense provided by the plant’s resident microbiota.

One model system that has emerged for the reductionist study of plant immunity and microbial plant protection is the *Pseudomonas syringae–Arabidopsis* pathosystem [4, 17, 18]. *Pseudomonas syringae pv.* Tomato DC3000 (*Pto*) is a Gram-negative bacterial leaf pathogen that infects a variety of plant species, including *Arabidopsis* [18]. *Pto* is a valuable model for dissecting plant–pathogen interactions, leading to key findings into the functioning of the plant’s immune system, and more recently for examining plant microbiome–pathogen interactions. Bacterial isolates, primarily from the phyllosphere, have been studied for their potential to inhibit the growth and virulence of *Pto* either directly [19], or by induced resistance such as ISR [20]. For example, beneficial rhizobacteria have been found to produce volatile organic compounds that trigger a defense response against *Pto* in *Arabidopsis*, resulting in reduced disease severity [21, 22]. Another study demonstrated that the beneficial rhizobacterium *Pseudomonas simiae WCS417* stimulates plant growth and induces ISR against a broad range of pathogens, including *Pto* [23].

Recently, large culture collections were screened for protective effects against *Pto* infection in controlled model systems. These studies revealed a widespread potential for plant protection among phyllosphere bacteria from across bacterial phylogeny [4, 24]. Follow-up studies, using *Sphingomonas* and *Rhizobium* insertional loss-of-function mutants, identified several bacterial cellular processes as involved in plant protection against *Pto*, including respiratory chain functions, regulatory processes, cell shape maintenance, and type VI secretion systems [25, 26]. These studies were often reduced binary plant—microbe axenic systems, in which the plant is cultivated devoid of any other microbes except the one under investigation. This setup enables researchers to observe the specific impacts of a particular microbe on the plant, without the interference of other microorganisms [27]. However, both the ecological and translational insights that may be gained from such systems are limited by the fact that diverse communities of interacting species naturally colonize plants and the effects of single microbial species on the host are not always additive. A recent study addressed this issue by applying a set of randomly selected SynComs and using machine learning to identify the best combinations of protective bacteria [28], but the many thousands of microbial taxa inhabiting plants offer an impossibly enormous number of combinations to test for.

Here, we tested a new screening strategy for ecologically relevant traits within the microbiome, that we term “top-down community deconstruction.” In this approach, we begin our screen with a large SynCom, and iteratively drop-out different taxonomic groups to identify emergent plant phenotypes. This allows for the identification of phenotypes that are manifested in the presence of multiple interacting species. Within this context, we aimed to understand how the root microbiome affects disease outcomes of leaf pathogens and how the ecological context of the microbiome determines plant phenotypes when facing disease, asking which key members of the root microbiome are important for improving plant tolerance to the leaf pathogen *Pto*. We inoculated plant roots with a complex root-derived SynCom and with taxonomic subsets of this SynCom, examining the effect on leaf colonization by the pathogen. We demonstrate that removing only the genus *Bacillus* from the microbial community allows the pathogenic bacterium *Pto* to colonize *Arabidopsis thaliana* leaves, demonstrating a crucial role for *Bacillus* in plant protection.

## Materials and methods

### Bacterial culture and plant inoculation

The 150-member bacterial synthetic community (SynCom) used here contains genome-sequenced isolates (Supplementary Dataset S1) obtained from surface-sterilized Brassicaceae roots, nearly all *A. thaliana*, planted in two soils from North Carolina (USA). A detailed description of this collection and isolation procedures can be found in ref. [29]. The phylogenetic trees used here are subsets from the single-copy gene tree used in ref. [29].

One week before each experiment, bacteria were inoculated from 50% glycerol stocks at −80°C into 500 μl Luria Bertani (LB) medium in a 96-deep-well plate. Bacterial cultures were grown at 28°C, shaking at 250 rpm. After 4 days of growth, cultures were inoculated into fresh medium and returned to the incubator for an additional 48 h, resulting in two copies of each culture (6 days old and 48 h old). This method was chosen to account for the varied growth rates of the bacterial strains and to ensure that the inoculum included nonstationary cells from each strain. The optical density of each one of the individual cultures was measured at 600 nm (OD_600_) using an Infinite 200 Pro plate reader (TECAN). Bacterial cells were combined based on the optical density measurements into single tubes according to phylum. Then, the optical density of the new tubes was measured at 600 nm (OD_600_). All cultures were pooled while normalizing each culture, before and after pooling, to OD_600_ = 0.1, centrifuged at 4000 rpm for 15 min at 4°C, the supernatant was removed and the pellet was resuspended in 5 ml of liquid Murashige–Skoog (MS) medium [30]. MS medium was prepared according the following formula: 2.2 g MS (plantMedia), 0.5 g MES, 1 l double-distilled water, pH adjusted to 5.7 with 1 M KOH.

The resuspended solution was then diluted to OD_600_ = 0.1 (~1 × 10^7^ colony forming unit [CFU]/ml) for inoculation in different experimental systems:

**Closed gnotobiotic system**: Coconut coir was placed in Magenta GA-7 culture boxes (plantMedia) and subjected to three rounds of gamma irradiation, 25 kGy each. The lid was removed in a sterile hood, 30 ml of sterile liquid MS medium was added to the coir inside magenta boxes, and plants were placed in these boxes. Then, 5 ml of the bacterial suspension was added to each magenta box. The boxes were then covered with a transparent breathable membrane (Breathe-Easy sealing membrane).
**Agar system**: The resuspended bacterial solution was applied to the MS agar at OD_600_ = 0.0001 (~1 × 10^5^ CFU/ml), 2 ml were added to each well in the plate.
**Open system**: 40 ml of liquid MS medium was added to the coir inside planting boxes, and plants were placed in these boxes. The bacterial solution was diluted to OD_600_ = 0.5 (~5 × 10^7^ CFU/ml). Then, 1 ml of the suspension was added to the designated planting boxes.

### Seed sterilization and plant growth conditions

*Arabidopsis thaliana* seeds (ecotype Columbia; Col-0) were surface sterilized with a cleaning solution (10% bleach and 0.1% Tween-20), shaken for 15 min at 1000 rpm, followed by five rinses with sterile distilled water. Seeds were stratified at 4°C for 24–48 h in the dark. After stratification, seeds were transferred to square plates containing MS medium supplemented with 0.8 agar and 1% sucrose. Plates were kept in a vertical position for 7 days in a growth chamber under a 16-h light/8-h dark regime at 21°C day/18°C night. On the 8th day, the resulting seedlings were transferred to magenta boxes containing 250 ml soil sterilized three times at 25 kGy of gamma radiation. This soil was watered with 30 ml MS medium. In the agar system, the resulting seedlings were transferred to 12-well plates. Each well contains 2 ml of MS medium supplemented with 0.55% agar. Upon collection, DNA was extracted from roots, shoots, and the agar substrate. Here and hereafter, all measurements were taken from distinct samples.

### DNA extraction

Roots, shoots, and rhizosphere were collected separately, pooling five plants for each sample. Roots and shoots were placed in 2-ml Eppendorf tubes with three sterile glass beads. These samples were washed three times with sterile distilled water to remove soil particles and weakly associated microorganisms. Tubes containing the samples were stored at −80°C until processing. Root and shoot samples were lyophilized for 48 h. DNA extractions were carried out on ground root and shoot tissue using DNeasy PowerSoil Pro Kits (Qiagen) following the manufacturer’s instruction. Sample position in the DNA extraction was randomized, and this randomized distribution was maintained throughout library preparation and sequencing.

### Bacterial 16S rRNA gene sequencing

The V3–V4 regions of the bacterial 16S rRNA gene were amplified using primers 338F (5′-ACTCCTACGGGAGGCAGCA-3′) and 806R (5′-GGACTACHVGGGTWTCTAAT-3′). Two barcodes were added to the 5′ end of 338F, and to the 3′ end of 806R primers. PCR conditions were as follows: 2 μl Phusion GC buffer, 0.2 μl dNTP, 0.3 μl DMSO, 0.1 μl Phusion Polymerase, 0.5 μl 338F (10 μM, 0.5 μl 806R (10 μM), 0.5 μl mixed of oligonucleotide-based on a locked nucleic acids (LNA) of plastid and mitochondrial rRNA (10 μM), 5.4 μl water, and 0.5 μl Template DNA. Temperature cycling: 98°C for 30 s, 30 cycles of 98°C for 10 s, 78°C (LNA) for 15 s, 55°C for 15 s, 72°C for 20 s, 4°C until use. The PCR product was diluted 1:100. The PCR product was indexed using 96 indexed 806R primers with the same reaction mix as above and 10 cycles of cycling conditions. PCR products were purified using AMPure XP magnetic beads and quantified with a Qubit 2.0 fluorometer (Invitrogen, Carlsbad, CA). Then, samples were multiplexed with Nextera dual indexes and sequenced on a MiSeq system (Illumina) using a 600-cycle V3 chemistry kit. We used the QIIME2 pipeline to process the sequencing output with default settings and the Greengenes2 16S rRNA gene reference database for taxonomic assignments [31]. The QIIME2 output was then used for producing figures in R using the phyloseq package [32, 33]. All relevant code is found at https://github.com/Amal-Gh-Mh/Bacillus-protection.code2-12-24.

To assess the contribution of seed endophytic microbiome or of potential contamination during the DNA extraction and amplification process, five control samples (blanks and gamma-irradiated coir) were included and processed under the same conditions as all other samples. These controls were run on an electrophoresis gel and subjected to sequencing. No bands were detected. We added these samples to the sequencing runs and did not identify any amplicon sequence variants (ASVs) there that contaminated the other samples.

### Infection with *P. syringae Pto*-lux

The *luxCDABE*-tagged *Pto* was obtained from Detlef Weigel’s lab (Max Planck Institute, Germany). The bioluminescent strain of *Pto* was made by the insertion of the *luxCDABE* operon from *Photorhabdus luminescens* into the *Pto* chromosome under the control of a constitutive promoter. Bacterial fitness, plant development, or disease outcome were unaffected by the integration of *luxCDABE* [34]. The bioluminescence of *Pto* was used as the indicator of pathogen colonization, enabling easy distinction from the SynCom bacteria added to the system. Bacteria were streaked from a −80°C glycerol stock onto liquid LB medium and grown for 24–48 h at 28°C. The culture was centrifuged at 4000 rpm for 15 min, and the culture supernatant was washed with 10 mM MgCl_2_. The OD_600_ of the suspension was adjusted to 0.05. In the agar system, the OD_600_ of the suspension was adjusted to 0.3 and the suspension diluted to a final OD_600_ of 0.00003. The plants were infected by spraying bacterial suspension in 10 mM MgCl_2_ with 0.03% Silwet L-77 (Union Carbide).

### Quantification of bacteria using colony forming units

The bioluminescent bacterial colonization analysis was used to score the SynCom’s protection of the plants. After 6 days of infection with *Pto*, shoot samples were removed from infected *Arabidopsis* plants and placed in a sterile tube with glass beads and 200 μl of 10 mM MgCl_2_. For the experimental validation in unsterilized coir (Fig. S3C), leaf samples were surface sterilized at this point by immersing them in 70% ethanol. Plants were crushed using two cycles of 30 s at high speed, in a Mini-Beadbeater (Biospec). Samples were serially 1:10 diluted six times in 10 mM MgCl_2_, and this dilution series was plated on LB plates, with 4 μl per spot. The plates were placed at 28°C for ~2 days and then the CFUs for each dilution of each sample were counted. To determine the *Pto-lux* bacterial population in inoculated leaf tissue, photos of luminescent bacteria were taken after 48 h of growth on LB plates by a high ISO (52 000) camera (Sony a7 III ILCE7M3) with an exposure time of 30 s.

### Halo assay

To scan the SynCom members for inhibition of the pathogenic bacterium *Pto*, we co-cultured each one of the SynCom bacteria with a *Pto* lawn on agar plates. For bacterial inoculants, SynCom strains and *Pto* were inoculated from a −80°C glycerol stock into liquid LB medium and grown overnight. The OD_600_ of the *Pto* culture was adjusted to 0.1 and 100 μl were spread on the LB agar plate. The SynCom strains were diluted to OD_600_ = 0.1 in LB and 4 μl were spotted on the top of the *Pto*. The plates were placed into an incubator at 28°C. Photos of the plates were taken after 24 h of growth on LB plates by a high ISO (~52 000) camera (Sony a7 III ILCE7M3) with an exposure time of 30 s. ImageJ (FIJI) software [35] was used to analyze luminescence levels at ten points from the center of the bacterial colony, providing an estimate of color strength based on a black-to-blue gradient determined by the software.

### Quantification of plant phenotypes

To evaluate the effects of the SynCom bacteria on healthy plant phenotypes, and the impact of pathogenic bacteria, ImageJ (FIJI) software [35] was used to measure the shoot area. The color pixel counter module within FIJI was used to score plant disease symptoms. The color pixel counter module within FIJI quantified disease symptoms by analyzing color differences associated with infected regions, associated with either water-soaked lesions, or dead tissue. To calculate the percentage of diseased area, the symptomatic area was divided by total shoot area. We imaged bacterial luminescence on leaves using the IVIS Spectrum optical imaging system (Revvity, USA). In the IVIS-based pixel analysis, individual pixel counts for each color were multiplied by the corresponding values from the IVIS software’s color scale, and the totals were summed to obtain a final disease severity measurement.

### Measuring *Bacillus* leaf colonization

To measure the ability of our *Bacillus* strains to reach the shoot from soil, we grew *Arabidopsis* seedings in unsterilized coir. We inoculated soil planted with 1-week-old seedlings by pipetting directly into the soil, avoiding contact with the leaves, 2 ml washed culture of either a mixture the of 22 *Bacillus* strains included in the SynCom, or green fluorescent protein (GFP)-labeled *Bacillus subtilis* NCBI 3610 (amyE::_PrrnE_-gfp) [36] at an OD_600_ = 0.5. For CFU counts, leaf samples from plants inoculated with the 22 *Bacillus* strains, or not, were collected after 2 weeks, weighed, and placed in a sterile tube with glass beads and 200 μl of 10 mM MgCl_2_ and then plated for CFU counts as described above. After 24 and 48 h of growth, we performed colony PCR using the 16S rRNA gene primers, on all of the colonies in the countable dilutions (we consider 5–100 colonies per droplet as countable). PCR products were sequenced using Sanger sequencing and identified using the SILVA ACT service. For microscopy, after 3 weeks, shoot samples inoculated or not with *B. subtilis* were removed from infected *Arabidopsis* plants and washed in MgCl_2_, vortexing the tube for 15 s. The leaves were then removed, and the leaf wash was centrifuged at 6800 *g* for 1 min. Most of the supernatant were removed, and 20 μl of the resuspended pellet was mounted on a slide and visualized on an inverted Nikon ECLIPSE Ti2 microscope, with the following parameters: Calibration: 0.11 μm/px; Objective: PLAN APO λD 40× OFN25 Differential Interference Contrast (DIC) N2; Modality: WF Fluorescence, BRF. DIC and GFP channels are shown separately and combined [37]. Only CFUs with 16S rRNA gene sequences matching the *Bacillus* at the genus level were counted.

### Soil sterilization test before the start of the soil experiments

An enrichment method was used to check the sterility of soil treated with three rounds of gamma irradiation (25  kGy per cycle). Five milliliter of soil was mixed with 25 ml of liquid LB medium in a sterile Erlenmeyer flask. The flask was covered and placed into an incubator at 28°C, with the shaker set to 168 rpm. Every 3 days the culture was refreshed with 1:2 LB liquid medium. This refreshment was done twice. Additionally, every day of the incubation, 100 μl of the enrichment culture was plated on LB agar plates for bacterial detection, and the plates were placed for incubation at 28°C for 1–3 days.

### RNA extraction

Three replicate samples of plant RNA were collected from the shoots of each treatment. The samples were washed with sterile water to remove soil particles, placed in 2 ml Eppendorf tubes with three sterile glass beads, and flash-frozen in liquid nitrogen. Tubes were stored at −80°C until processing. RNA was purified from plant tissue using the RNeasy Plant Mini Kit (Qiagen, Hilden, Germany) according to the manufacturer’s instructions and stored at −80°C.

### RNA sequencing

Illumina-based RNA-Seq libraries were prepared from 2 μg RNA. Plant mRNA was purified from total RNA using Sera-mag oligo (dT) magnetic beads (GE Healthcare Life Sciences) and then fragmented in the presence of divalent cations (Mg^2+^) at 94°C for 6 min. The resulting fragmented mRNA was used for first-strand cDNA synthesis using random hexamers and reverse transcriptase, followed by second-strand cDNA synthesis using HiFi DNA polymerase I and RNaseH. The DNA fragments were then adenylated to allow the ligation of Illumina KAPA adapters. Following library preparation, quality control and quantification were performed using an Agilent 2100 Bioanalyzer-nano chip, and library quantity and pooling were measured by Qubit (dsDNA HS). Libraries were sequenced using NextSeq 500 high output kit V2 75 cycles to generate 30 million single-end reads per sample.

### Processing of RNA-Seq reads

Initial quality assessment of the RNA-Seq reads was performed using FastQC. Trim galore was used to remove the first 10–12 bases from the 5′ end of the reads. The resulting high-quality reads were then mapped against the TAIR10 *Arabidopsis* reference genome using the Rsubread package [38]. The featureCounts function was used to count reads that mapped to each one of the nuclear protein-coding genes. Based on principle component analysis, one outlier sample was removed from each treatment. DESeq2 was provided to identify genes that are differentially expressed between different conditions or groups.

### Quantification and statistical analysis

All experiments were done in at least three biological repeats. Measurements were done in either technical duplicates or triplicates. The statistical analysis was conducted using RStudio with R 3.6.3. A linear regression model, specifically the “lm” function, was employed to analyze the data. To assess the differences between each pair of treatments, a Tukey’s Honestly Significant Difference (HSD) test was performed at a confidence level of 95% using the “TukeyHSD” function. The “generate_label_df” function was utilized to extract the labels and factor levels from Tukey’s post hoc results. Compact letter displays were then generated to represent the significant differences among the treatment groups. The default settings were applied, assigning the same letter to groups with no significant difference. This methodology facilitated the identification and visualization of significant differences between the treatment groups.

### Data resources

The underlying data for all figures in the manuscript are included as Supplementary Dataset S2. The 16S rRNA gene amplicon sequencing data associated with this study have been deposited in the NCBI Sequence Read Archive under the project accession PRJNA1175571. The raw transcriptomic data have been deposited in the Gene Expression Omnibus under the accession GSE285599.

## Results

### Establishment of a plant–microbiome model system

To investigate plant microbiome–plant–pathogen interactions, we constructed a closed model system consisting of *A. thaliana* Col-0 plants (hereafter Arabidopsis) grown in gamma-irradiated coconut coir within magenta boxes, covered with a transparent breathable membrane. We destroyed the resident microbes in the coir, by subjecting it to a high dose of gamma irradiation (3 × 25 kGy). To ensure that the coir was indeed sterile, before each experiment, we made enrichment cultures of a coir sample in LB broth and plated samples from this enrichment culture during a week-long incubation. We did not observe any microbial growth (Supplementary Fig. S1A). We replaced the destroyed microbiota with a 150-member SynCom composed of bacterial isolates previously retrieved from *Arabidopsis* roots [1] (Supplementary Fig. S1B and Supplementary Dataset S1). Plants growing in the irradiated coir were severely growth impaired compared with plants grown in non-irradiated coir, but the introduction of the SynCom to the irradiated coir partially restored plant growth (Supplementary Fig. S1C). To measure SynCom colonization of the plant, we extracted DNA from inoculated rhizosphere, roots, and shoots of 3-week-old plants and performed 16S rRNA gene amplicon sequencing. We found that 100% of the 16S rRNA gene reads in our data originated from the inoculum, confirming that the resident microbiota was destroyed and completely replaced by our SynCom (Supplementary Dataset S3). Soil, root, and shoot samples assembled distinct communities (Supplementary Fig. S1D), recapitulating the habitat partitioning observed in natural plant microbiomes [1, 2]. Therefore, our growth system, consisting of gamma-irradiated coconut coir protected with a breathable membrane, is a useful model for microbiome reconstruction experiments and measuring microbial effects on plants.

### The genus *Bacillus* was identified as a focal taxon in enhancing plant defense

**Figure 1:**
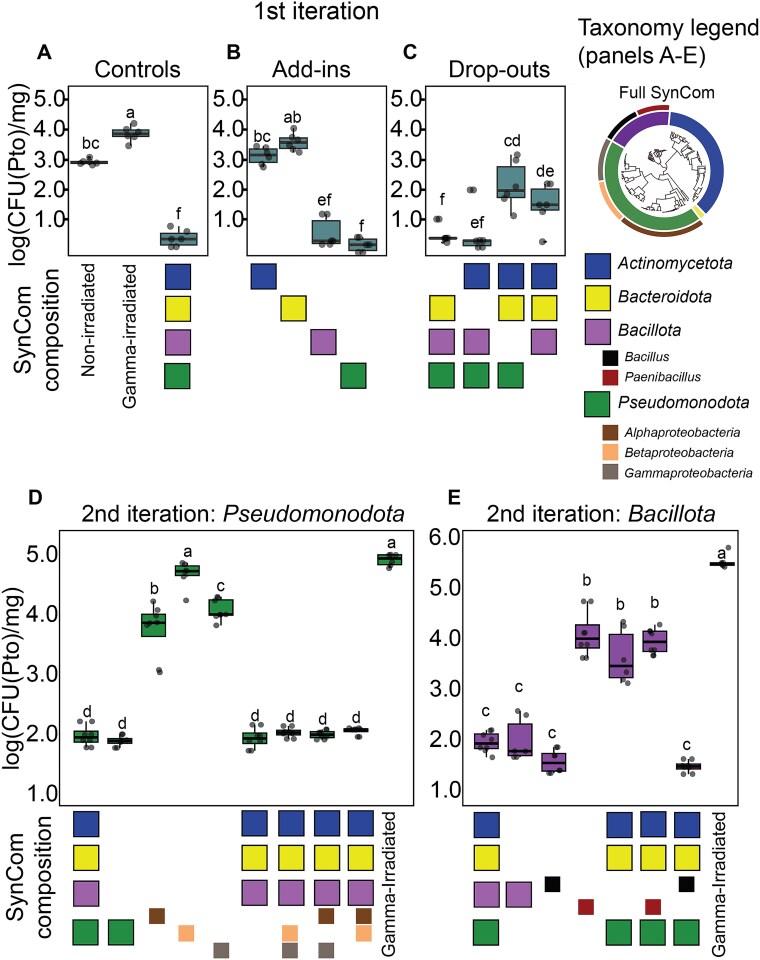
Luminescent CFU measurements of *Pto* at the top-down community deconstruction experiments. (A) Plants grown without the SynCom, in both irradiated and non-irradiated substrate, compared to plants grown with the complete SynCom on irradiated substrate. (B) Plants treated with each phylum separately. (C) Plants grown with four drop-out treatments, removing each bacterial phylum, separately, from the inoculum. Statistical analysis was conducted for panels (A–C) collectively; significance was determined via ANOVA, and letters indicate compact letter display from a Tukey post hoc test. (D) Second iteration of top-down community deconstruction within *Pseudomonadota* phylum, inoculating the plant with each *Pseudomonadota* class separately (add-in) or removing each class from the inoculum (drop-out). Significance was determined via ANOVA, and letters indicate compact letter display from a Tukey post hoc test. (E) Second iteration of top-down community deconstruction within *Bacillota* phylum, inoculating the plant with either of the *Bacillus* or *Paenibacillus* groups separately (add-in) or removing each genus from the inoculum (drop-out). Significance was determined via ANOVA; letters correspond to a Tukey post hoc test.

To quantify the protective effect of our soil-inoculated SynCom against a leaf pathogen, we infected the leaves of SynCom-inoculated plants with a lux-tagged *Pto* [34] and measured the resulting population size of *Pto* per milligram of leaf tissue, 6 days post-infection. We observed that the complete SynCom protected the plants compared to the controls (Fig. 1A). Plants grown without the SynCom, in both irradiated and non-irradiated coir, had a high CFU count of luminescent *Pto* colonies on their leaves, compared with plants grown in coir that was inoculated with the complete SynCom (Fig. 1A), demonstrating that microbiota sourced from, and inoculated onto soil and roots, contribute to protecting the plant’s aerial parts from pathogen colonization.

To tease apart the roles of different microbiota members in maintaining plant growth, we applied a methodology we call “top-down community deconstruction” (Supplementary Fig. S2). This approach involves inoculating plants with a complete SynCom, and then iteratively dropping out taxonomic groups, to assess their contribution to the phenotype in question. The SynCom contains four bacterial phyla: *Actinomycetota, Bacillota, Bacteroidota*, and *Pseudomonadota* (including *Alpha-, Beta*-, and *Gamma-Proteobacteria*) (Supplementary Figs S1B and S2). We inoculated the gamma-irradiated coir with different subsets of the complete SynCom, removing one of the four phyla composing it (drop-out), or adding each phylum to the substrate separately (add-in). Six days post-infection, plants inoculated with only *Actinomycetota* or only *Bacteroidota* strains exhibited a relatively high titer of *Pto* in the leaves, whereas plants inoculated with only *Bacillota* or only *Pseudomonadota* strains were nearly infection-free, similar to the effect provided by the complete SynCom (Fig. 1B). The drop-out treatments complemented these results. Dropping out *Actinomycetota* or *Bacteroidota* did not compromise plant protection, also yielding a *Pto* titer which was just as low as with the complete SynCom. In contrast, dropping out *bacillota* or *Pseudomonadota* resulted in the partial loss of protection. In this case, the loss of protection was not complete, due to the fact that either *Bacillota* or *Pseudomonadota* were still present in either of these treatments (Fig. 1C). These results suggested that the plant microbiota requires the presence of populations from within the *Bacillota* and *Pseudomonadota* phyla to completely suppress leaf infection.

To identify the strains within both *Pseudomonadota* and *Bacillota* that are responsible for protecting the plant, we designed a second iteration of the drop-out experiment, in which we deconstructed both phyla. Within the *Pseudomonadota*, we separately dropped out each of the three classes *Alpha-, Beta-*, and *Gamma-proteobacteria*. None of these drop-outs compromised the protection provided by the SynCom (Fig. 1D). As this result could not resolve the role of *Pseudomonadota* classes, we also inoculated the plant with SynComs composed of each of the classes separately. In this case, alpha*-* and gamma-proteobacteria provided relatively minor protection, whereas betaproteobacteria did not protect the plant (Fig. 1D). This indicates that protection provided by *Pseudomonadota* depends on inoculum diversity, as one class alone does not confer protection, but a combinations of classes does.

Within the *Bacillota* phylum, we dropped out each of the two genera composing it: *Bacillus* and *Paenibacillus*. In this case, the removal of the *Bacillus* strains from the complete SynCom resulted in loss of protection, whereas removal of the *Paenibacillus* strains had no effect on protection (Fig. 1E). In agreement with this result, a SynCom composed of only *Bacillus* strains also provided complete protection, whereas a SynCom composed of only *Penibacillus* strains did not (Fig. 1E). All-in- all, in every combination where the inoculum included *Bacillus* strains, the leaves were protected from *Pto*, whereas in every combination that did not include *Bacillus* strains, protection was impaired, demonstrating that the genus *Bacillus* is a focal taxon in plant protection from *Pto*.

### *Bacillus* protect plants from *Pto* on the background of a natural microbiota

To test the applicability of our findings with the background of a naturally assembled community, we setup an experiment in an open plant growth system using non-irradiated coir. We designed three different treatments for this experiment: plants inoculated with a SynCom composed of all 22 *Bacillus* strains, plants inoculated with a SynCom composed of 22 strains of *Actinomycetota*, a phylum that did not show any protective effect in the irradiated coir system, and plants maintained with only the native coir microbiota.

**Figure 2:**
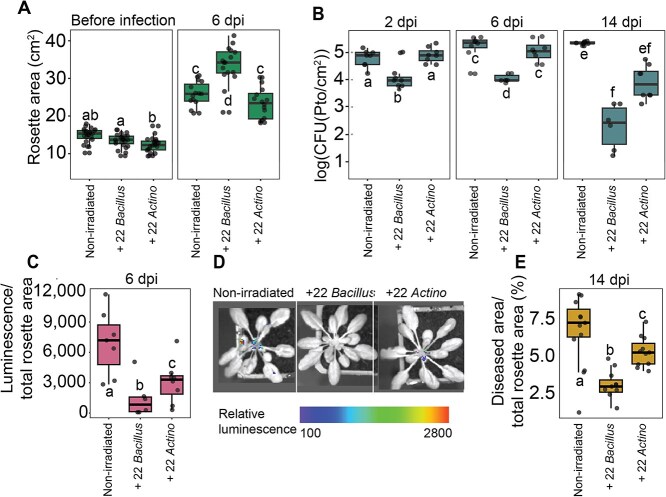
*Bacillus* strains protect plants from *Pto* in an open system. (A) The measurements of the rosette area were conducted on three treatments: Plants with the addition of *Bacillus* strains to the soil, plants with the addition of strains from the *Actinomycetota* phylum, and plants grown in non-irradiated soil with its natural microbiota at 2 time points: Two weeks before the infection (before infection), and after 6 days of infection (6 dpi). Significance was determined via ANOVA, and letters indicate compact letter display from a Tukey post hoc test. (B) The luminescent CFU measurements of *Pto* were conducted in the same treatments at three time points post-infection: 2 days (2 dpi), 6 days (6 dpi), and 14 days (14 dpi). Significance was determined via ANOVA, and letters indicate compact letter display from a Tukey post hoc test. (C) Luminescence level per total area measured by the IVIS Spectrum imaging system at the same treatments as in (C), 6 days post-infection (6 dpi). Significance was determined via ANOVA, and letters indicate compact letter display from a Tukey post hoc test. (D) Images from the IVIS Spectrum imaging system . Luminescence levels are indicated using artificial colors. (E) The measurements of the diseased area per total area of the plant at the same treatments, 14 days post-infection (14 dpi). Significance was determined via ANOVA, and letters indicate compact letter display from a Tukey post hoc test.

Three weeks into the experiment, plants inoculated with *Bacillus* strains showed better growth compared to those inoculated with *Actinomycetota* strains and exhibited similar growth to plants with the natural coir microbiota. Six days post-infection with *Pto*, the plants inoculated with *Bacillus* strains had larger rosette areas than those growing with the natural microbiota alone (Fig. 2A). We conducted CFU counts of *Pto* at three-time points: 2-, 6-, and 14-day post-infection. Plants inoculated with the *Bacillus* strains consistently showed lower CFU counts of *Pto* across all time points. As the experiment progressed, the *Pto* CFU counts of *Bacillus*-treated plants further decreased, underscoring the protective efficacy of the *Bacillus* community even amidst natural microbial communities (Fig. 2B). These findings were corroborated by directly measuring luminescence *in planta* and by measuring disease area per total shoot area (Supplementary Figs 2C–E and S3A). To ensure that the presence of *Bacillus* in the inoculum protects the plants not only from casual encounters with *Pto* but from bona-fide infection, in an additional experiment, we focused only on *Pto* entering the apoplast: using similar inoculations as above, we extracted disks from the plants and sterilized their surfaces to quantify only the bacteria within the leaf, excluding those on the outer surface. In this experiment as well, plants with the addition of *Bacillus* strains were larger in both infected and non-infected plants (Supplementary Fig. S3B). We detected luminescent *Pto* colonies solely in treatments devoid of *Bacillus* strains, with no luminescent CFU found in the *Bacillus*-treated group (Supplementary Fig. S3C). There were also significant differences in the percentage of diseased area per total rosette area between the treatments, highlighting the protective and beneficial effects of *Bacillus* strains on the plants (Supplementary Fig. S3D). Taken together, these results demonstrate the effectiveness of using a diverse consortium of root-derived *Bacillus* strains to protect plants from pathogen colonization, and that this effect is robust to different biotic backgrounds.

### All *Bacillus* strains inhibit *Pto in vitro*

**Figure 3:**
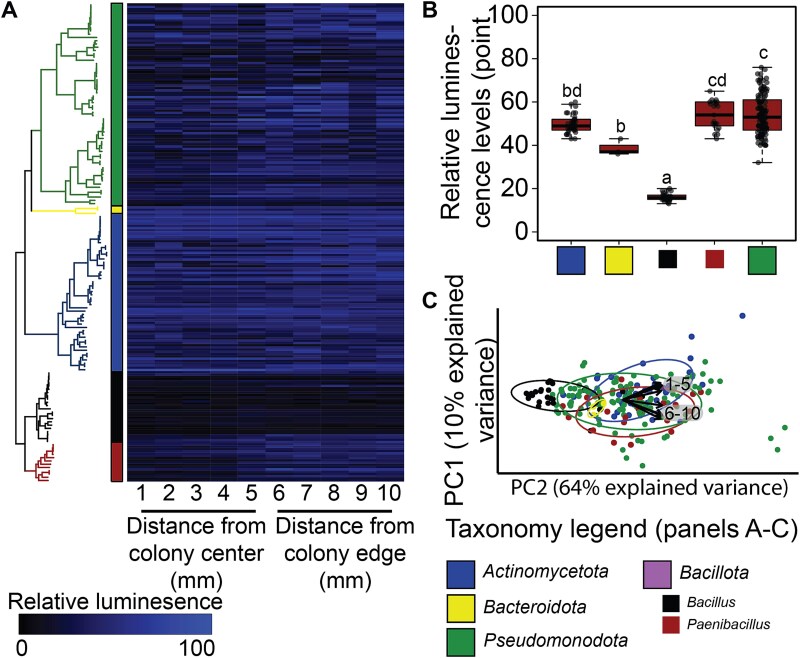
*Bacillus* strains inhibit *Pto* growth *in vitro*. (A) A heatmap depicting the luminescence levels across ten points. Strains are organized based on their phylogenetic tree, shown to the left of the heatmap. (B) Luminescence levels at point eight (~3 mm outside the inhibiting colony), exhibited the least variance within each phylum, after splitting the *Bacillota* phylum. Significance was determined by ANOVA, with letters indicating Tukey post hoc group differences. (C) A PCA plot illustrating the luminance levels at ten points within and outside of the colony for each SynCom bacterium. Each dot represents one strain from the SynCom circled and colored based on its phylum. Loading vectors are shown for the 10 points. See Fig. S4 for illustration of the position of the points relative to the colony.

The top-down drop-out approach that we applied, implicated *Bacillus* as a focal taxon in plant protection. This led us to ask which of the 22 *Bacillus* strains in our collection are responsible for the plant protection phenotype? In order to address this question, we tested the interaction between each member of the SynCom and *Pto* individually, on agar plates without plants present, using an inhibition halo assay. We performed co-inoculations by placing a droplet from a culture of each member of the SynCom on the surface of a *Pto* culture that was spread on agar plates. We modified the traditional halo assay, by measuring luminescence rather than looking for a clearing zone around the inhibitory colony, reasoning that this will make the assay more sensitive to the inhibitory effect. We measured *Pto* luminescence levels at 10 points, along a line starting from the center of the colony of each SynCom strain, with five points within the colony (representing inhibition under direct contact) and five points outside of it (representing diffusible inhibition at a distance from the colony) (Supplementary Fig. S4). All *Bacillus* strains exhibited the highest level of inhibition against *Pto*, with a strong inhibition halo both in direct contact and as far as 5 mm from the colony (Fig. 3A and B). Among *Pseudomonadota* strains we observed mixed results, with some strains inhibiting *Pto* only in close proximity (points 1–5), some inhibiting also at a distance (points 6–10) and some showing no inhibitory phenotypes. *Actinomycetota, Bacteroidota*, and *Paenibacillus* strains, for the most part, did not inhibit *Pto* (Fig. 3A and B).

To classify the interactions between the different stains and *Pto* in an unsupervised manner, we performed a principal component analysis (PCA) on the 10 luminescence values measured for each strain (Fig. 3C). The five points within the colonies (1–5) and the five points adjacent to the colonies (6–10) had an almost orthogonal contribution to the variance, indicating independence between direct and diffusible inhibition. As expected, *Bacillus* strains cluster separately from the rest of the SynCom strains (Fig. 3C), demonstrating their shared ability to inhibit *Pto* activity. Although most of the *Pseudomonadota* strains clustered separately from *Bacillus* strains, some exhibited similar behavior to the *Bacillus* strains. These results suggest that direct antagonism by *Bacillus* strains and some *Pseudomonadota* strains plays a role in plant protection from *Pto*.

### *Bacillus* inoculated in the soil translocate to the shoot

To antagonize *Pto* in the leaves directly, *Bacillus* strains would have to translocate from the soil in which they were inoculated, to the leaves. To measure the capability of *Bacillus* strains to translocate from soil to leaves, we inoculated soil planted with 1-week-old *Arabidopsis* seedlings with the consortium composed of our 22 *Bacillus* strains and measured their abundance on leaves 2 weeks following inoculation using CFU counts. We observed an average of 1e3.9 *Bacillus* CFUs per gram leaf in plants inoculated with the 22 *Bacillus* strains, whereas no *Bacillus* CFUs were detected on the leaves of uninoculated plants (Supplementary Fig. S5A). To directly observe *Bacillus* populations on leaves, we inoculated an additional set of pots, with a GFP-fluorescent strain of *B. subtilis* NCBI 3610 [36]. We inspected leaf washes under the microscope, 3 weeks post-inoculation, and observed the presence of fluorescent *Bacillus* colonies (Supplementary Fig. S5B). The cells we observed in the leaf washes were in colony form, with elongated cells, suggesting that *Bacillus cereus* forms biofilms on leaf tissue (Supplementary Fig. S5B). These results support the notion that *Bacillus* strains are able to translocated from the rhizosphere to the leaves, where they can directly engage with a leaf pathogen such as *Pto*.

### *Bacillus* strains harbor unique and diverse antimicrobial activity genes

As the *Bacillus* strains all inhibited *Pto in vitro* and produced an inhibition halo indicative of a diffusible compound, we hypothesized that the *Bacillus* strains in our collection share a common biosynthetic gene cluster (BGC) producing a compound that inhibits *Pto*. In order to identify genes potentially involved in producing such a compound, we applied the BGC search tool AntiSMASH to all of the genomes in the SynCom [39]. This tool identified a diverse repertoire of BGCs among this collection of plant-associated bacteria, including among the *Bacillus* strains (Supplementary Fig. S6A and B). For example, several of the *Bacillus* genomes encode production of *Bacillus* lipopeptides (Supplementary Dataset S4), which were previously implicated as important in plant protection from a broad range of pathogens [40]. However, we could not identify any BGCs that were common to all *Bacillus* strains and absent in the other strains in the collection. This indicates that contrary to our initial hypothesis, *Bacillus* strains likely act via a number of distinct mechanisms or via similar mechanisms, using distinct molecules.

### *Bacillus* strain diversity is essential for protection

**Figure 4:**
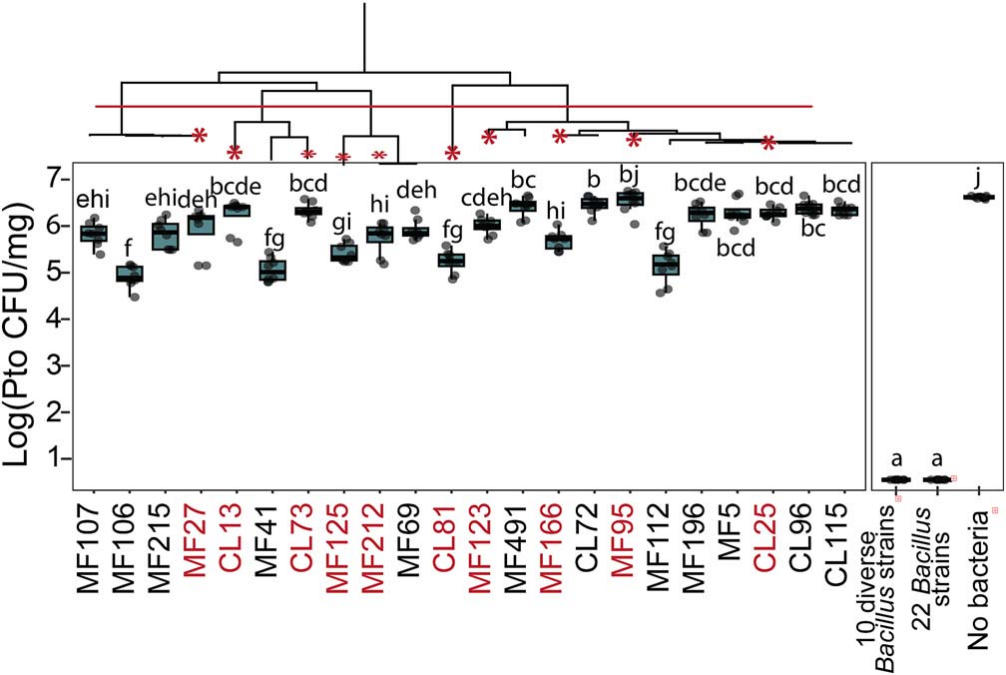
Synergistic effects of diverse *bacillus* strains on plant development and health. (Top) Phylogenetic tree of *bacillus* strains, highlighting the top 10 strains representing the greatest diversity in red stars. (Bottom) Luminescent CFU measurements of *Pto* in plants inoculated with individual strain from 22 *Bacillus* strains. Controls included all ten diverse *bacillus* strains, as well as groups with all *bacillus* strains and those without any bacteria. Letters indicate significant differences based on Tukey’s post hoc test.

We have established (i) that in these experimental conditions the presence of *Bacillus* strains is necessary and sufficient to provide the plant with protection, (ii) that each one of the *Bacillus* strains individually inhibits *Pto* growth but (iii) that a common BCG could not be identified. This led us to hypothesize that the 22 different *Bacillus* strains in the SynCom provide protection through complementary interactions, rather than a conserved mechanisms, and that as such, their effect would be additive. To test this, we asked whether inoculation with a single strain would be sufficient to provide protection. We selected a set of 10 *Bacillus* strains from our collection that represent the highest possible phylogenetic diversity (Fig. 4). We grew *Arabidopsis* seedlings in gamma-irradiated coir, inoculated with each one of these 10 *Bacillus* strains separately, as well as a combination of all 10. Even before *Pto* infection, plants inoculated with each strain separately exhibited poor growth, indicating that the presence of only one bacterium in the soil is not sufficient for optimal plant development. In contrast, we observed normal plant growth when all 10 strains were added together (Supplementary Fig. S7). This finding demonstrates the importance of a diverse microbiota even within the genus level, but it precluded our ability to test the effect on pathogen success. We therefore repeated the experiment using a sterilized agar system, in 12-well plates. This system allows plants to grow well even in the absence of any bacteria added, providing a baseline for comparison. Using this system, we infected plants with *Pto* in the presence of each of the 22 *Bacillus* strains separately or in combination. Six days post-inoculation, we observed that plants inoculated with a combination of ten diverse *Bacillus* strains, as well as those with all 22 *Bacillus* strains included, showed no detectable *Pto* CFUs. In contrast, plants inoculated with individual *Bacillus* strains exhibited reduced, yet still substantial levels of *Pto* colonization compared to the control group of uninoculated plants (Fig. 4). These findings indicate that a single strain of *Bacillus* alone is not sufficient to provide complete protection to the plants from *Pto* infection.

### *Bacillus* strains act independently of immune system activation and compensate for its absence

**Figure 5:**
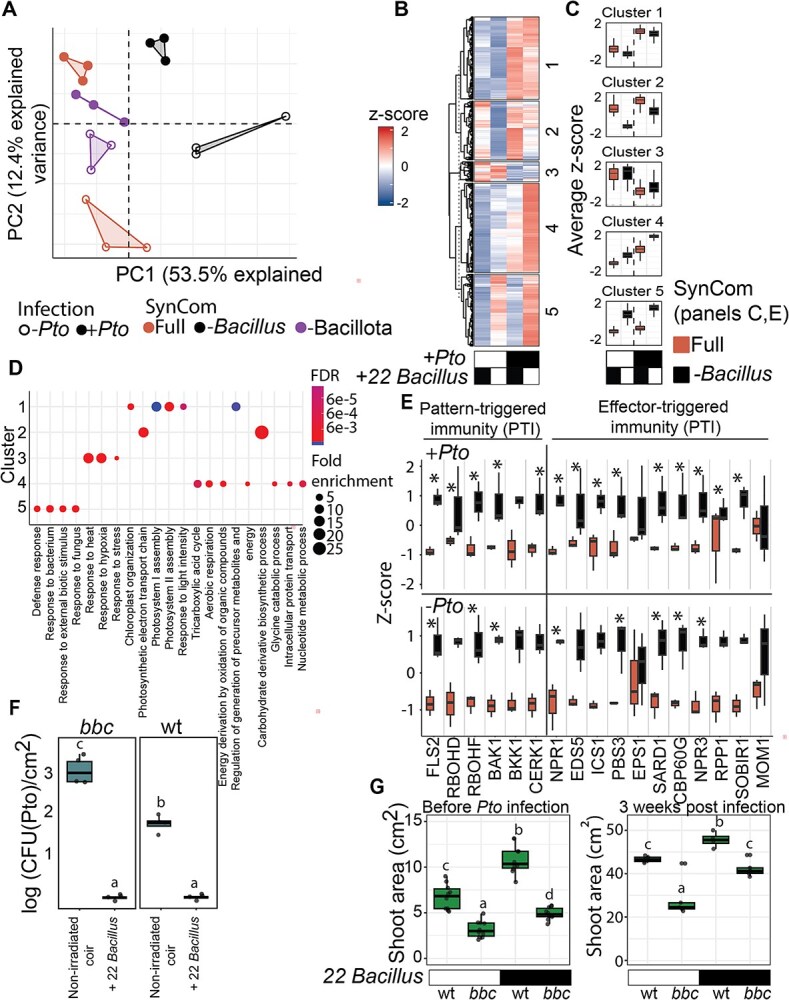
Gene expression analysis of plants with full SynCom and *Bacillus* drop-out under uninfected and *Pto*-infected conditions. (A) PCA of gene expression data, illustrating sample separation based on *Pto* infection (*Pto*-infected [filled shape] versus non-infected [empty shape]) and SynCom treatment (full-SynCom, -*Bacillus*, and -*Bacillota*). (B) Heatmap displaying gene expression profiles for full SynCom and *Bacillus* drop-out across five identified clusters, with *Z*-score normalization. (C) Box plots of expression levels within each cluster, emphasizing differential gene expression patterns according to *Pto* infection status (±) and SynCom composition. (D) Top gene ontology (GO) term enrichment for each co-expressed cluster, showing distinct biological processes. Circle color gradient represents the false discovery rate (FDR), and circle size reflects fold GO enrichment, with larger circles indicating a higher fold-change for each GO term relative abundance within the cluster compared with the entire transcriptome (e.g. greater enrichment). (E) Gene expression related to plant immune mechanisms—pattern-triggered and effector-triggered immunity of uninfected plants (top) and *Pto*-infected plants (bottom) in the two treatments: Full SynCom and *Bacillus* dropped out. Asterisks indicate significance based on the complete differential expression model. (F) CFU measurements of *Pto* in wt and *bbc1* mutant with and without the soil inoculation with 22 *Bacillus* strains. Letters indicate significant differences based on Tukey’s post hoc test. (G) Plant shoot area before (left) and 3 weeks after (right) Pto infection of wild type and *bbc* plants inoculated, or not, with 22 *Bacillus* strains in the soil. Letters indicate significant differences based on Tukey’s post hoc test.

To understand if the plant’s immunity plays a role in its protection by *Bacillus* strains, we investigated how the presence of *Bacillus* strains in the soil affects the expression of immune system genes in plants. We performed RNA-seq on the leaves of plants treated with different SynCom configurations, specifically comparing the full SynCom, *Bacillota* dropout, and *Bacillus* dropout groups in both uninfected and *Pto*-infected plants. The PCA plot reveals distinct transcriptional profiles based on SynCom composition and infection status (Fig. 5A). Focusing on the *Bacillus* drop-out treatments, we clustered the differentially expressed genes into five modules of co-expressed genes, each typical of a different combination of *Pto* and/or *Bacillus* inoculation (Fig. 5B and C). To understand which transcriptional modules were affected by the bacterial treatments, we performed gene ontology (GO) term enrichment analysis across the different clusters. Cluster 1, representing genes upregulated in the presence of *Pto*, showed significant enrichment in GO terms associated with photosystem assembly and degradation and with photoinhibition response, presumably reflecting damage to photosynthetic tissue caused by the infection to the leaves, and significant depletion in abiotic stress response genes. Cluster 2, representing genes upregulated in the presence of *Pto* but downregulated by the presence of *Bacillus* strains, was enriched in genes related with photosynthetic assembly but also in another group of genes belonging to the GO term “Glycine metabolic process.” Cluster 3, representing genes downregulated in the presence of *Pto* was enriched in genes related to response to abiotic stress, perhaps due to prioritization of biotic versus abiotic stress response. Cluster 4 represents genes that are upregulated additively by the presence of *Pto* and *Bacillus* strains. Genes belonging to this cluster were weakly enriched among several GO terms related with metabolisms and respiration. Cluster 5, representing the plant’s response to the presence of *Bacillus* strains in the microbiota, shows significant depletion in biotic stress response and defense response categories, indicating dampening of plant defense in this presence of *Bacillus* strains (Fig. 5B and D).

Focusing on defense response, we examined marker genes involved in both pattern-triggered immunity and effector-triggered immunity [41]. We observed a consistent general trend of induction of these genes in the *Bacillus* drop-out treatment compared to the full SynCom treatment (Fig. 5E). This suggests that the presence of *Bacillus* strains in the community reduced the expression of immune system genes. Together with our *in vitro* inhibition results (Fig. 3A–D), and with our observation that *Bacillus* strains translocate from the soil to the leaves (Supplementary Fig. S5), these results suggest that *Bacillus* strains protect plants from pathogens by direct antagonism while attenuating immune response.

To further disentangle immune response from direct inhibition, we tested whether the presence of *Bacillus* strains in the community also protects immunity-impaired plants. We used the *Arabidopsis* Pattern-recognition receptor co-receptor triple mutant bak1/bkk1/cerk1 (bbc1), which lacks the three major co-receptors that recognize bacteria-associated molecular patterns [42]. This mutant is hypersensitive to *Pto* infection [43]. Using an open system, with unsterilized coir, we compared *bbc1* plants inoculated with 22 *Bacillus* strains, to un-inoculated plants for their ability to withstand infection with *Pto*. Six days post-infection, among un-inoculated plants, we observed approximately one order of magnitude more *Pto* CFUs in the *bbc1* mutant than in wild-type leaves. *Bacillus*-inoculated plants from both genotypes were infection free (Fig. 5F). Three weeks post-infection, *bbc1* plants inoculated with the *Bacillus* consortium were grown to a comparable size to wild-type plants, which were only mildly affected by *Pto* (Fig. 5G). We concluded that inoculating the rhizosphere with a consortium of *Bacillus* strains compensates for the lack of a fully functional immune system, protecting the plant from a leaf pathogen.

## Discussion

In this work, by using an iterative drop-out approach, we were able to causally link a particular genus within the root microbiota—*Bacillus*, to protection from invasion by a bacterial leaf pathogen. *Bacillus* is a well-known genus for its biocontrol potential [44], which has been studied for decades [45, 46]. The *Bacillus* genus, in particular the model species *B. subtilis*, are known for producing a wide variety of antimicrobial compounds that are effective against numerous plant pathogens, enhancing plant growth and health [47]. For instance, studies have highlighted the ability of *Bacillus* strains to suppress fungal diseases and promote plant health through the production of bioactive compounds [48]. *Bacillus subtilis* was also shown to be effective against *Pto* on *Arabidopsis* roots, in a manner dependent on production of the cyclic lipopeptide surfactin [46]. Our work demonstrates that *Bacillus* strains inoculated in soil, can also be effective against *Pto* on leaves. We also show that *Bacillus* strains perform this critical plant protection role in an ecologically relevant context. A recent screen for biocontrol agents against *Pto* among phyllosphere bacteria, one-by-one, identified multiple highly protective strains, none of which belonged to the *Bacillota* phylum [49]. The different results among the two efforts can be explained by three important differences: the use of a different growth substrate (coconut coir vs. agar), the use of a different culture collection, sourced from different plant tissue (shoot vs. root) and the use of an opposite screening approach (bottom-up vs. top-down). The advantage of our top-down iterative deconstruction approach i.e. provides a scalable system for rapid identification of causal relationships in the plant microbiome and that the ecological context is maintained. Another advantage of SynCom deconstruction can be to study ecological interactions between the different members of the microbiota [50]. However, this was not the goal of the present study. While 16S rRNA gene amplicon sequencing confirmed successful colonization by the SynCom and revealed distinct microbial profiles in soil, root, and shoot compartments, we did not use this method to track individual strains in the community. In fact, many of the isolates in our SynCom share identical 16S rRNA gene sequences despite having distinct genomes, limiting strain-level resolution. Based on genome data, the SynCom is expected to correspond to ~60 unique 16S sequences; however, only 41 ASVs were detected in the sequenced inoculum, although viable cells were confirmed to be present for all strains. Several factors likely contribute to this underrepresentation, including differences in 16S rRNA gene copy number, DNA extraction efficiency, and amplification biases. Work with known inocula in soils exposes limitations and biases inherent to culture independent analyses, especially amplicon sequencing, illustrating that these data should be considered with caution, also when the inoculum is not known.

Several of the taxa for which we could not measure an effect under these conditions might have protective effects under different conditions than the ones used here. Moreover, we normalized the SynCom cultures in this based on OD_600_, but *Bacillus* strains have a relatively high ratio between viable cell counts and OD_600_. As a result, the concentration of the average *Bacillus* strains inoculated in the SynCom experiments was 3.2 × 10^7^ CFU/ml, ~1/15th of the average OD_600_ across the full SynCom. The *Bacillus* strains were able to overcome this low initial abundance and protect the plant.

*Bacillus* spp. can form endospores, allowing them to survive in harsh environmental conditions, making them particularly useful in agricultural settings where reliability and effectiveness over time are crucial [51]. Much of the research on *Bacillus* strains as biocontrol agents is driven by proven activity *in vitro*, ease of culturing, and ease of formulation, because *Bacillus* form durable endospores. This raises the concern that the convenience of using *Bacillus* strains biases the research toward this genus, overstating its importance. The outcome of field application of *Bacillus* strains a biocontrol agent has indeed been varied [47]. Our investigation showed that the protective effect depends on the diversity of the inoculum. Only when multiple *Bacillus* strains were present together were the leaves protected from infection. This synergy highlights the importance of microbial diversity in enhancing plant protection. The importance of diversity of biocontrol agents was demonstrated in other systems as well [52]. For instance, the diversity of *Pseudomonas* species inoculated into soil was positively correlated with biocontrol of *Ralstonia solanacaerum* [53]. These finding should be carefully considered when attempting to select bacterial inocula for plant protection, supporting the design of suppressive consortia rather than single strains.

The fact that bacteria isolated, then inoculated onto belowground plant parts, were active against a leaf pathogen, serves as a reminder that bacteria, and their influence, do not limit themselves to specific tissues (or plant species) that we may define for them and that the above- and belowground microbiota are in constant flux. Microorganisms in the root compartment could influence shoot defense phenotypes remotely, via systemic effects in the plant, including, but not limited to ISR [12, 54], but microorganisms may also travel from the root to the shoot and induce localized effects [55]. Our results indicate that *Bacillus* strains, rather than inducing an immune response, dampen the expression of immune markers, which has the potential to preserve valuable energy for the plant, allowing it to divert it toward growth, as is evident by the growth resistance trade-off [56]. Although the isolates are inoculated into the rhizosphere, their effect on a leaf pathogen appears to be local, as viable *Bacillus* populations were observed on the plant leaves following inoculation. We therefore, suggest that *Bacillus* engages the pathogen directly, on the leaf, with minor involvement of plant immune response, although further research into the involvement of induced resistance is needed in order to fully tease apart induced immunity from direct pathogen inhibition.

*Bacillus* are not the only plant protectors within the microbiota. We also observed protection coming from *Pseudomonadota*. As *Pto* is a member of *Pseudomonadota* itself, we hypothesize that niche overlap plays an important role in the mode of protection in this case [53, 57]. In contrast to *Bacillus*, in the case of *Pseudomonadota*, we could not identify a focal taxon, as it appears that only combinations of bacteria from two or more classes within the phylum are effective. This could also be an indication for the importance of diversity, albeit at a wider taxonomic resolution. Improvements in predicting resource utilization by microbes will allow us to identify strong competitors to these pathogens and rationally design protective consortia [49]. *Bacillus* and *Pseudomonas* strains were shown to act synergistically in plant protection [58], supporting the notion that robust protective consortia may be designed by combining members of these two highly cultivable groups.

## Supplementary Material

Supplementary-material_wraf134

## Data Availability

All data generated or analyzed during this study are included in this published article and its supplementary information files. The sequencing data have been deposited in the NCBI BioProject repository under accession number PRJNA1175571, https://www.ncbi.nlm.nih.gov/bioproject/PRJNA1175571/.
