# Supplementary figures and images for: Diverse rhizospheric Bacillus are required for protection against a leaf pathogen
Source: ISME J. 2025 Jul 1;20(1):wraf134. doi: 10.1093/ismejo/wraf134 (PMC13235741; doi:10.1093/ismejo/wraf134)

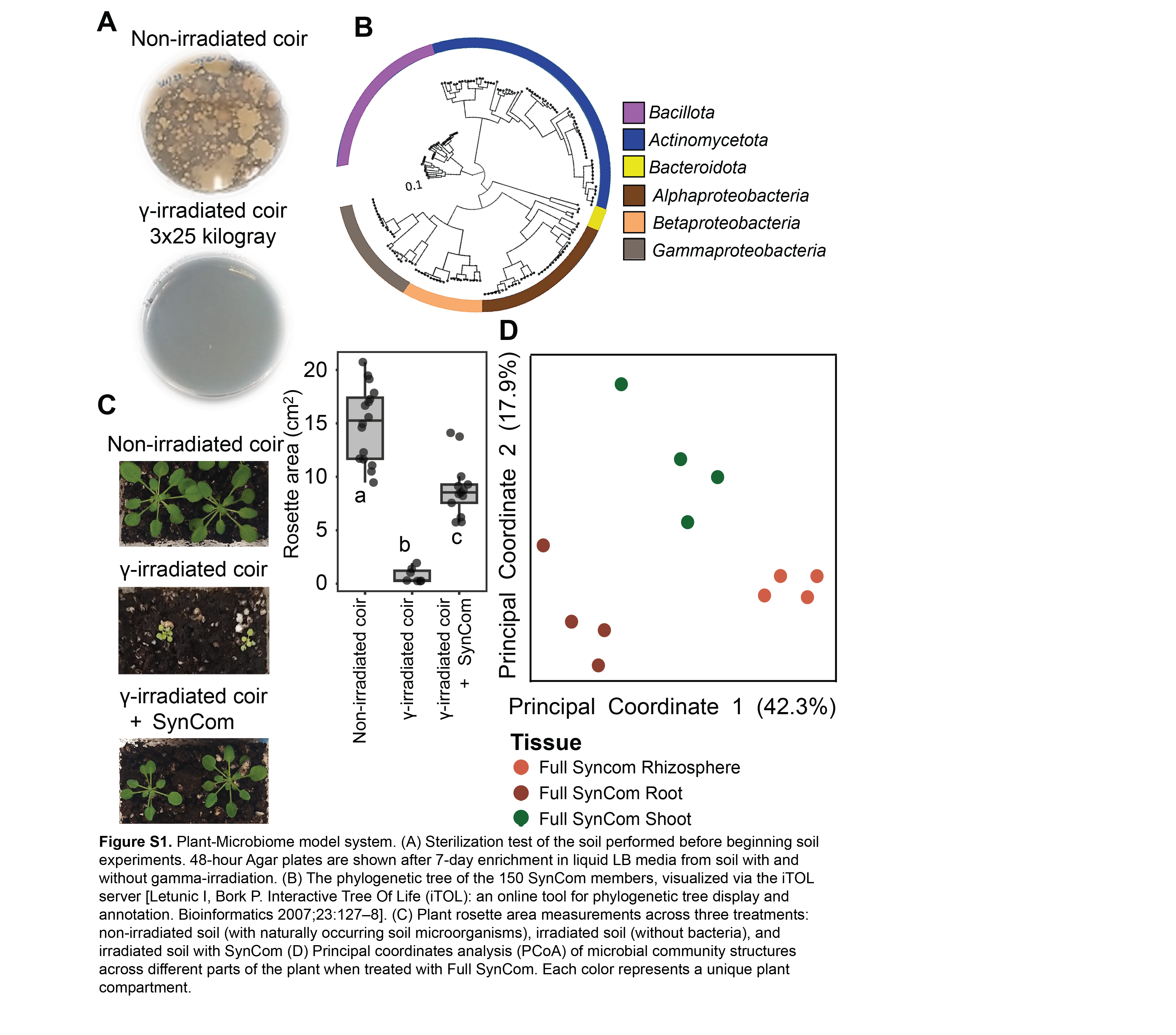

Supplement: Supplementary-material_wraf134 [file supplementary-material_wraf134.zip › S1.png]

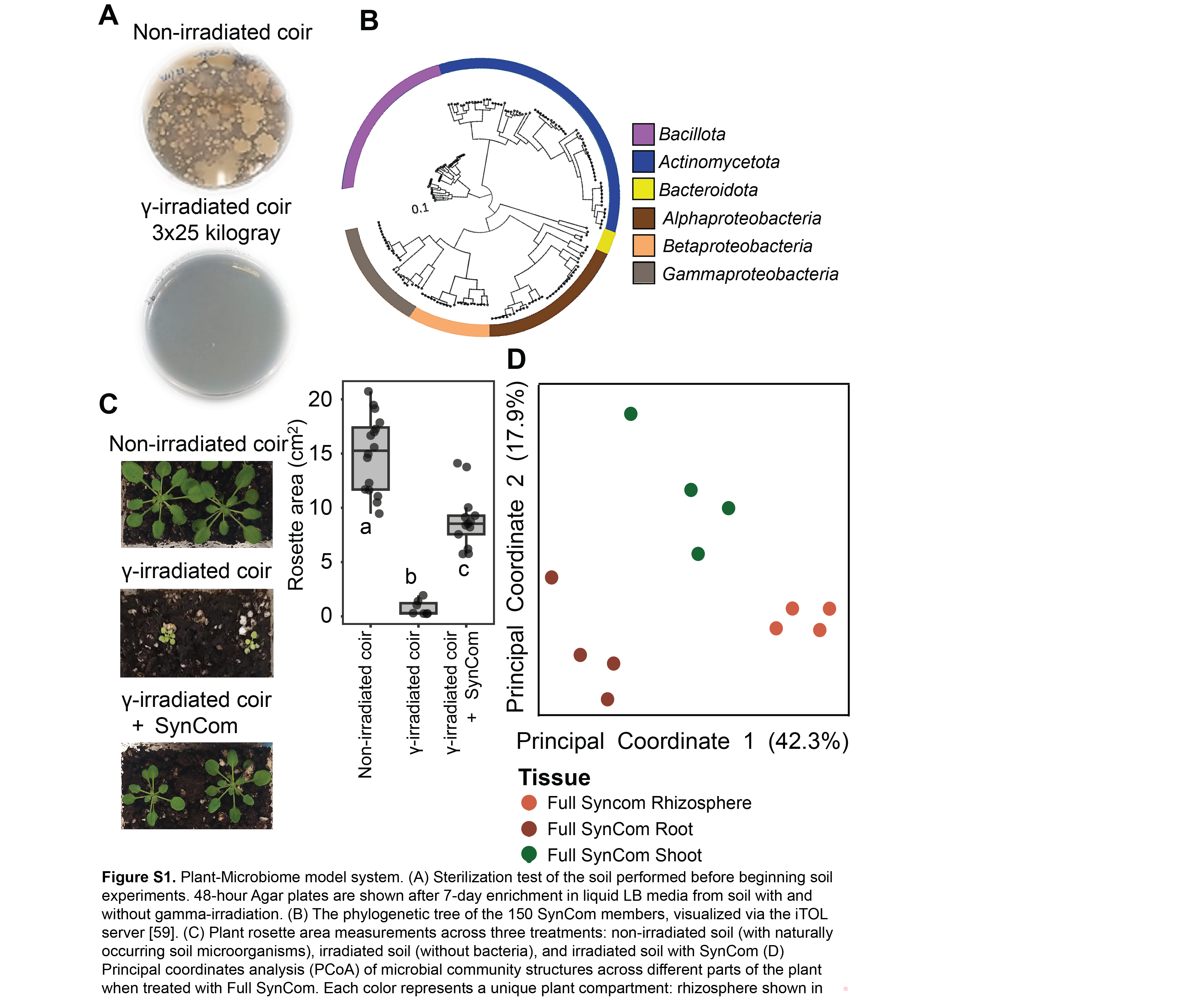

Supplement: Supplementary-material_wraf134 [file supplementary-material_wraf134.zip › S1_wraf134.png]

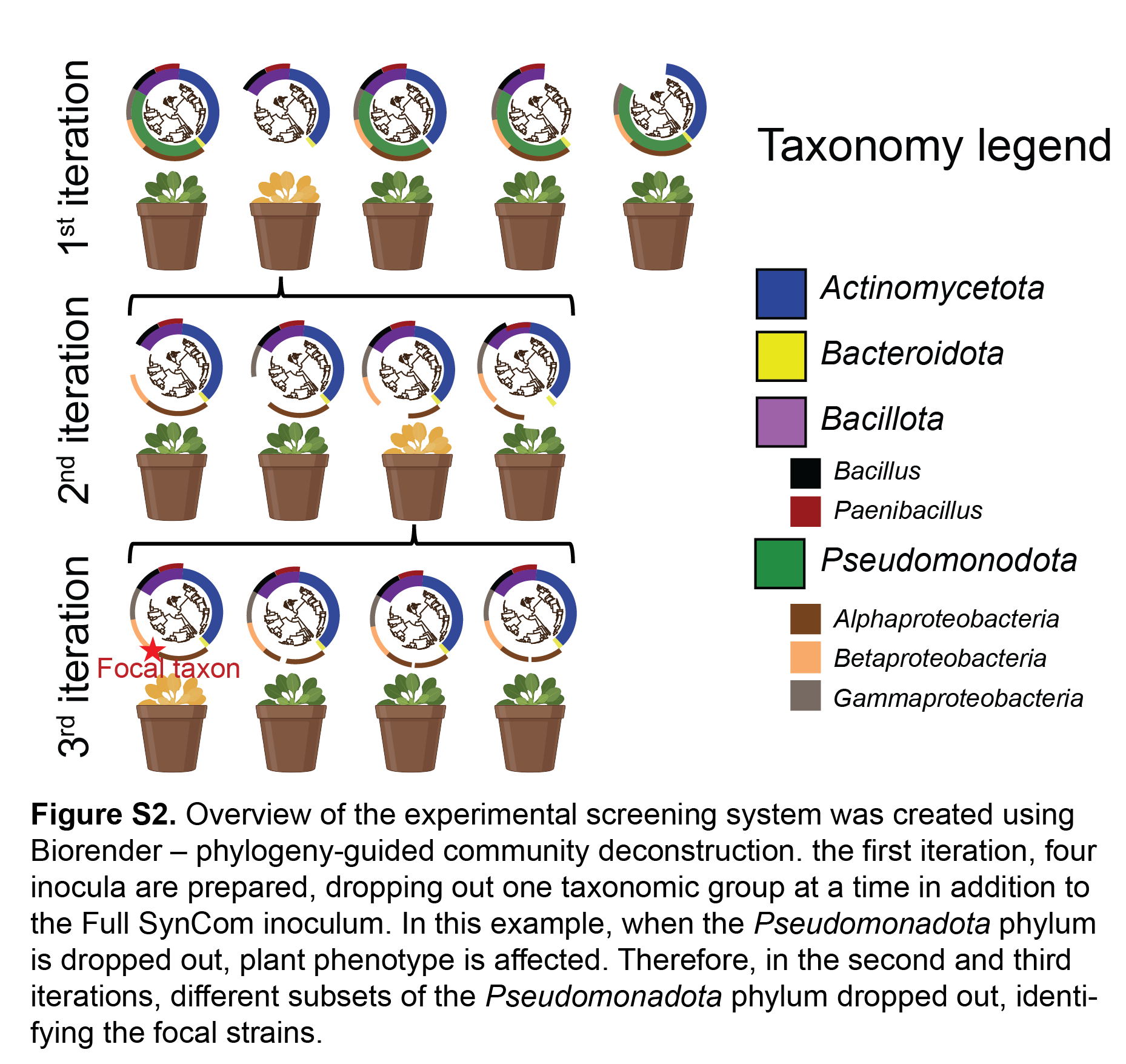

Supplement: Supplementary-material_wraf134 [file supplementary-material_wraf134.zip › S2_wraf134_new.png]

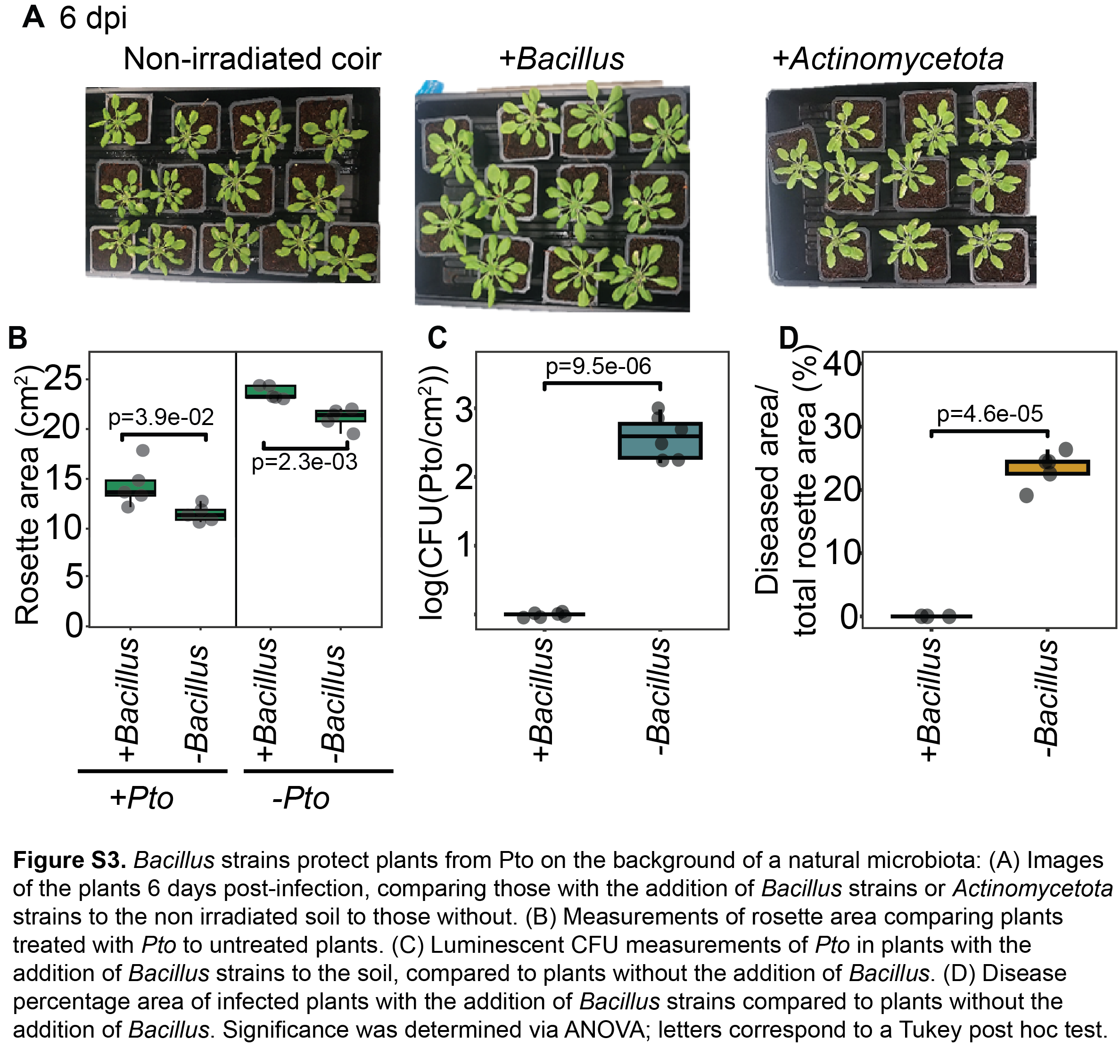

Supplement: Supplementary-material_wraf134 [file supplementary-material_wraf134.zip › S3_wraf134.png]

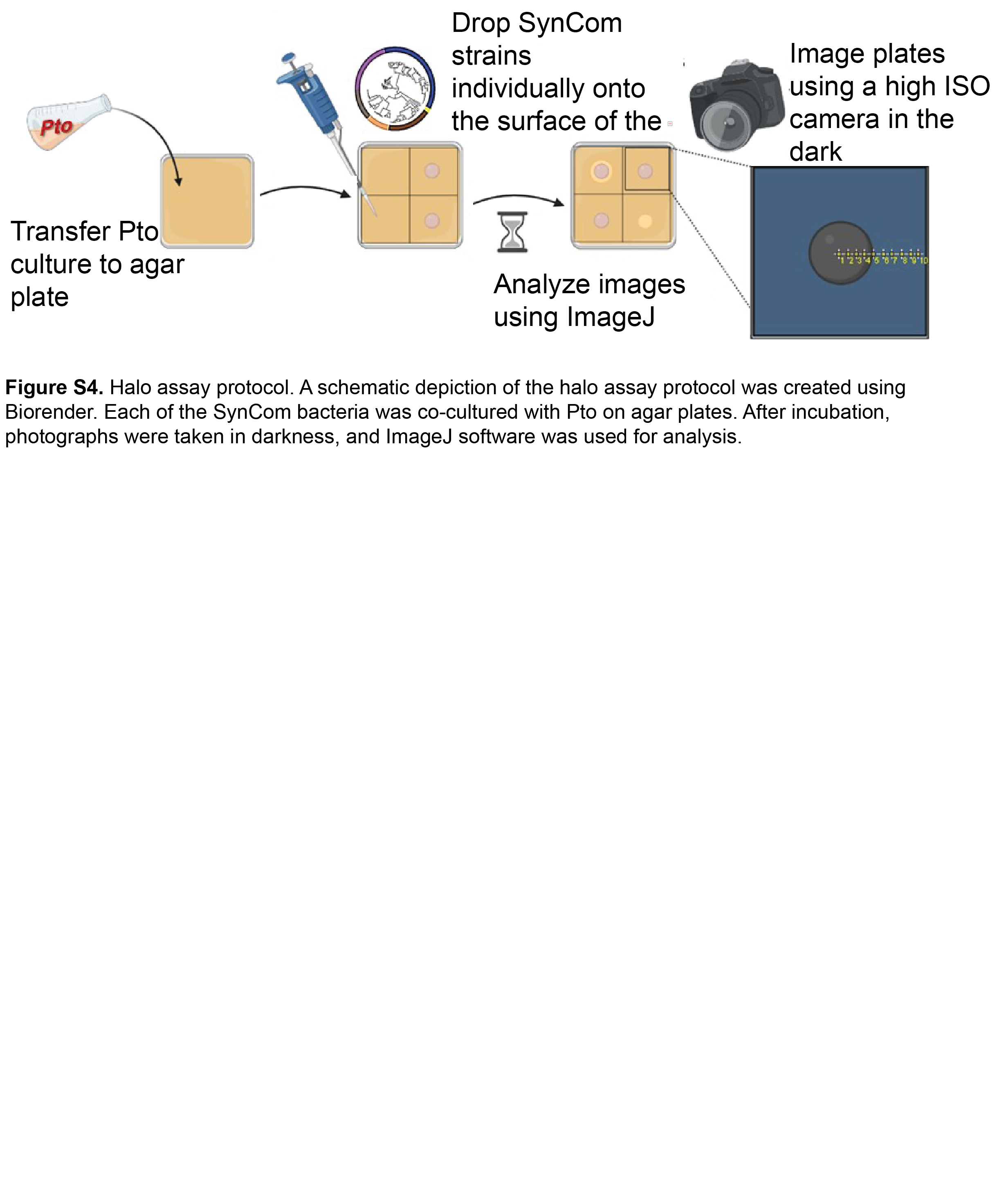

Supplement: Supplementary-material_wraf134 [file supplementary-material_wraf134.zip › S4_wraf134.png]

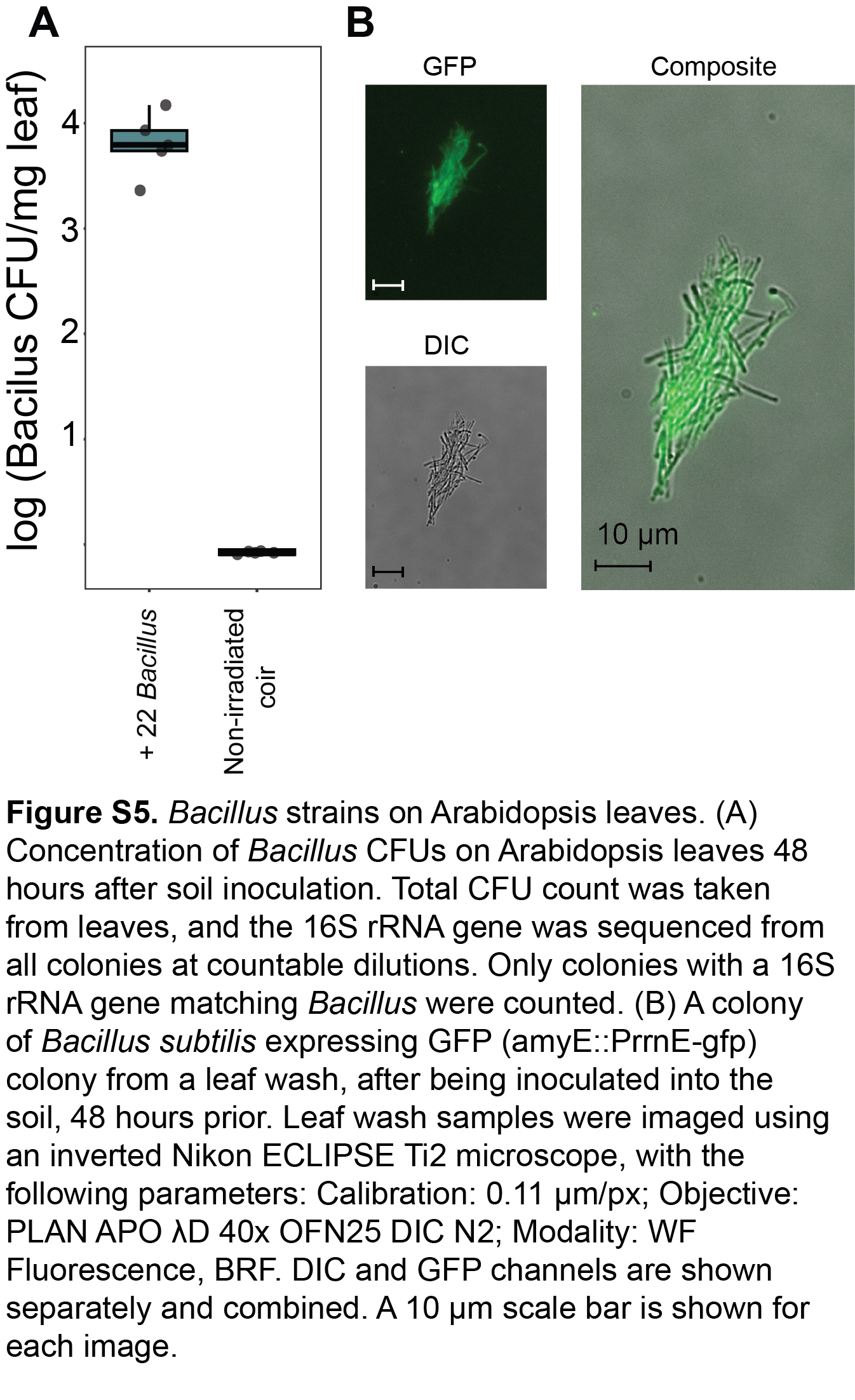

Supplement: Supplementary-material_wraf134 [file supplementary-material_wraf134.zip › S5ab_wraf134.png]

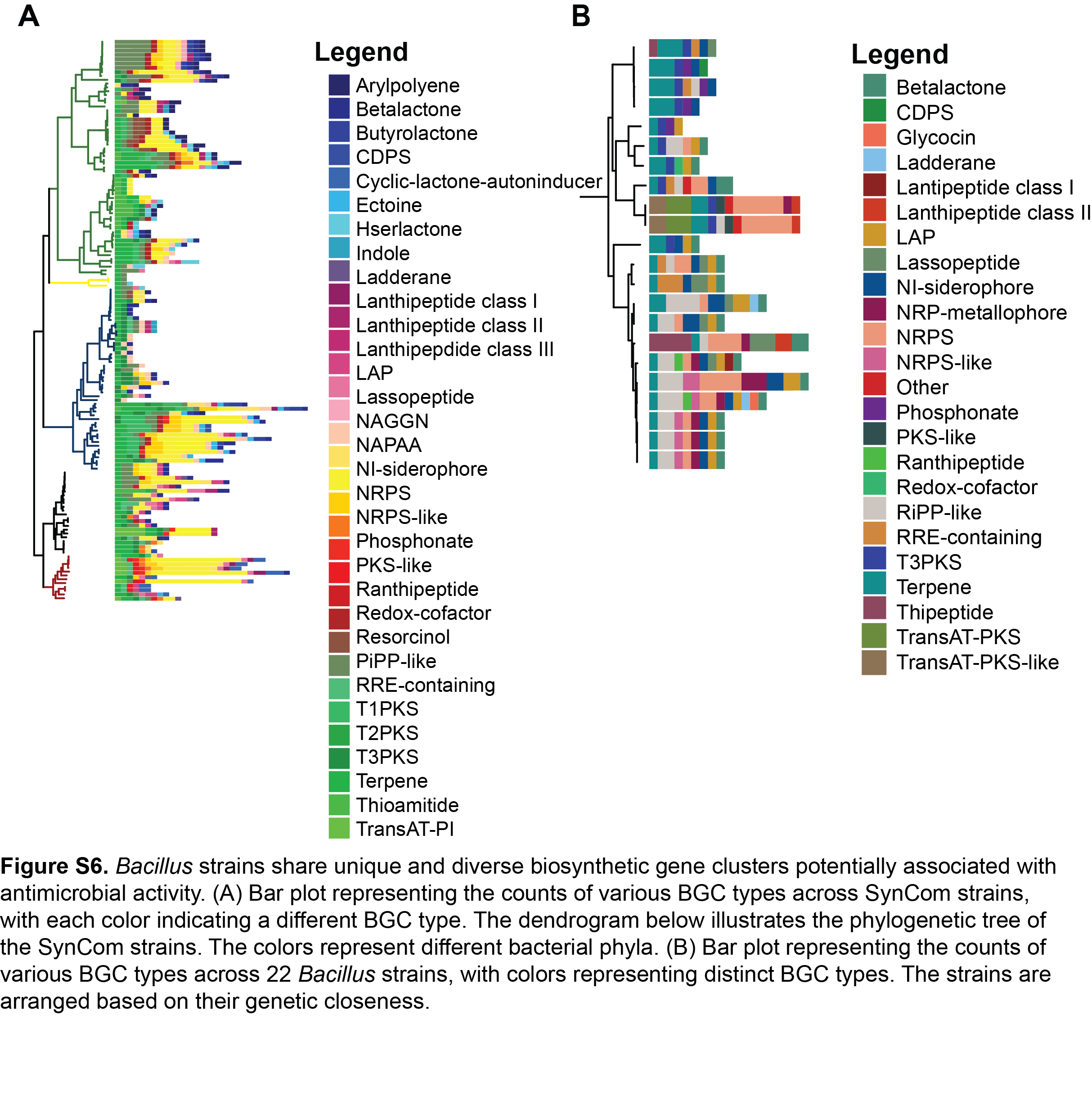

Supplement: Supplementary-material_wraf134 [file supplementary-material_wraf134.zip › S6_wraf134.png]

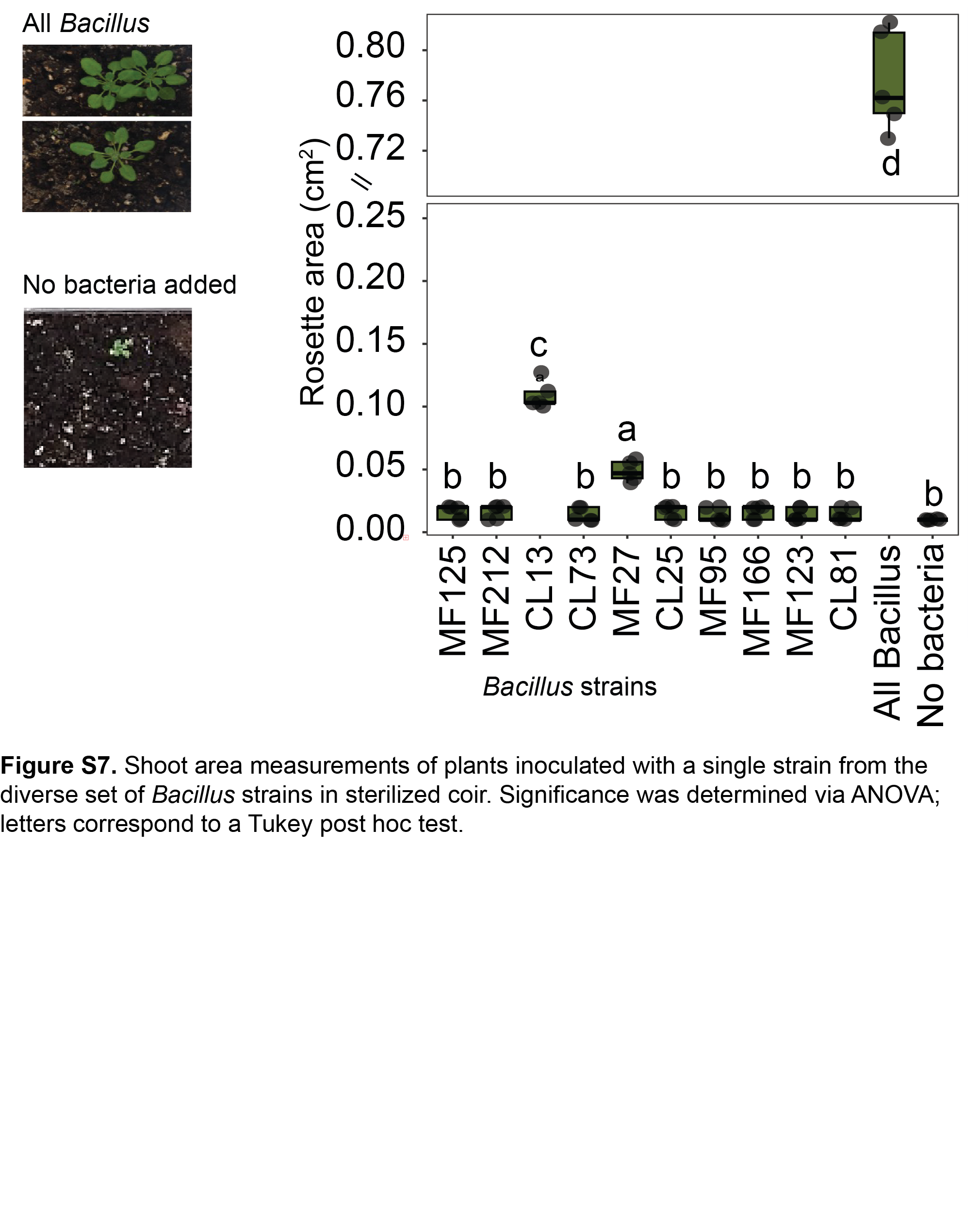

Supplement: Supplementary-material_wraf134 [file supplementary-material_wraf134.zip › S7_wraf134.png]
